# Effects of thermal treatment on the adhesion strength and osteoinductive activity of single-layer graphene sheets on titanium substrates

**DOI:** 10.1038/s41598-018-26551-w

**Published:** 2018-05-25

**Authors:** Ming Gu, Longwei Lv, Feng Du, Tianxiao Niu, Tong Chen, Dandan Xia, Siyi Wang, Xiao Zhao, Jianzhang Liu, Yunsong Liu, Chunyang Xiong, Yongsheng Zhou

**Affiliations:** 1 Department of Prosthodontics, Peking University School and Hospital of Stomatology, 22 Zhongguancun Avenue South, Beijing, 100081 PR China; 2 Department of Mechanics and Engineering Science, College of Engineering, Peking University, Beijing, 100871 PR China; 3 National Engineering Laboratory for Digital and Material Technology of Stomatology, National Clinical Research Center for Oral Disease, Beijing Key Laboratory of Digital Stomatology, Beijing, 100081 PR China

## Abstract

In recent years, dental implants have become the preferred approach for the restoration of missing teeth. At present, most dental implants are made of pure titanium, and are affected by peri-implantitis and bone resorption, which usually start from the implant neck, due to the complex environment in this region. To address these issues, in this study we modified the surface of titanium (Ti) implants to exploit the antibacterial and osteoinductive effects of single-layer graphene sheets. Chemical vapor deposition (CVD)-grown single-layer graphene sheets were transferred to titanium discs, and a method for improving the adhesion strength of graphene on Ti was developed due to compromised adhesion strength between graphene and titanium surface. A thermal treatment of 2 h at 160 °C was found to enhance the adhesion strength of graphene on Ti to facilitate clinical transformation. Graphene coatings of Ti enhanced cell adhesion and osteogenic differentiation, and imparted antibacterial activity to Ti substrate; these favorable effects were not affected by the thermal treatment. In summary, the present study elucidated the effects of a thermal treatment on the adhesion strength and osteoinductive activity of single-layer graphene sheets on titanium substrates.

## Introduction

Graphene, first isolated by Novoselov and Geim in 2004, has a unique two-dimensional (2D) hexagonal structure made of sp^2^-hybridized carbon atoms^1^. Since its discovery, graphene has attracted significant attention in various fields, owing to its excellent optical, mechanical, chemical, and electrical properties^2–5^. Further extensive studies have started to highlight the unique potential of graphene in the biomedical field, and increasing attention is being paid to its biomedical and biotechnological applications, such as biosensors, drug delivery, and tissue engineering^6–9^. Several studies have reported promising effects of graphene and its derivatives on cell adhesion, proliferation, and osteogenic differentiation^10–12^. In a previous study, we also confirmed that monolayer graphene could promote the osteogenic differentiation of human adipose-derived stem cells (hASCs) and human bone marrow-derived mesenchymal stem cells (hBMMSCs) *in vitro* and *in vivo*, and explored the epigenetic role of graphene in the fate of stem cells^13^. Moreover, the antibacterial effects of graphene have also been documented^14–16^.

Nowadays, dental implants have been widely used and largely improved the life quality of billions of patients who suffered from missing teeth^17,18^. However, the neck of a dental implant is the weakest area where infection and bone resorption begins. It is a challenged area because it emerges from the bone to the gingiva, and is in contact with a complex bacterial environment of the oral cavity. Therefore, bone integration, gingival attachment and anti-bacteria ability should be realized at the same time. However, better adhesion of osteoblasts, mesenchymal stem cells, or gingival fibroblasts, and less adhesion of bacteria is always contradicted^19^. It has been well recognized that rough surfaces are better for the cell adhesion^20–24^, but on the other hand facilitate the adhesion of bacteria^25^. On the other hand, smooth surfaces reduce the adhesion of bacteria^25^, but interfere the attachment of gingival fibroblasts and osteoblasts thus negatively influencing gingival attachment and osteogenesis^19,23,26^. Graphene appears as a possible solution to this contradiction, owing to its potential osteogenic and anti-bacterial ability. Single-layer graphene can be successfully produced in large scale by chemical vapor deposition (CVD) on metal, such as copper and nickel^27,28^. However, titanium is the most used material as medical implants^20,29,30^. The modification of the titanium surface with monolayer graphene can be solved by transferration using polymethyl methacrylate (PMMA)^3,31–33^. In our previous study, we found that the adhesion strength of graphene on the Ti surface is not satisfied enough for future clinical application^13^. Therefore, it is urgent to find a solution to improve the adhesion strength between graphene and titanium.

Thermal treatment is the last step of the transferring process to remove PMMA, and we found that prolonged time and higher temperature of thermal treatment improve the adhesion between graphene and Ti substrate. Therefore, in this study, we attempted to improve the adhesion strength of graphene on the surface of a Ti substrate through a thermal treatment. We investigated the effects of the graphene coating (with and without thermal treatment) on the *in vitro* and *in vivo* cell adhesion, proliferation, and osteogenic differentiation. In addition, the antibacterial effects of graphene (with and without thermal treatment) were also assessed. The incorporation of single-layer graphene is expected to impart a stable antibacterial and osteoinductive properties to the titanium surface.

## Results

### Surface characterization

**Figure 1:**
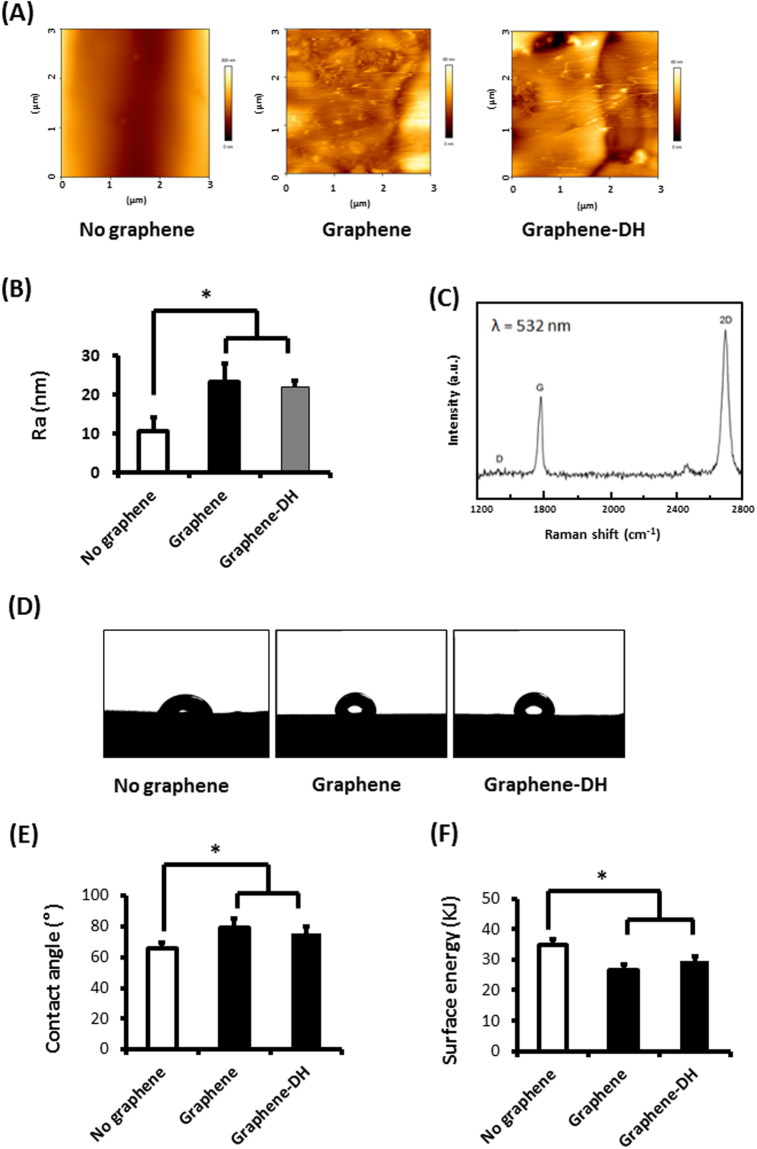
Surface characterization of graphene. (**A**) AFM images of graphene, graphene after dry heating treatment (Graphene-DH) and Ti (No-graphene) surfaces. (**B**) Roughness analysis of graphene, graphene after dry heating treatment and Ti surfaces. (**C**) Raman spectra of graphene surface. (**D**) Water contact angle images of graphene, graphene after dry heating treatment and Ti surfaces. (**E**) Water contact angle. (**F**) Surface energy. *P < 0.05.

The AFM images in Fig. 1A showed that the single-layer-graphene-coated Ti presented a much rougher morphology, compared with the smooth surface of No-graphene (uncoated Ti). And the roughness of graphene-coated Ti was higher than uncoated Ti (Fig. 1B).

Raman spectroscopy (Fig. 1C) demonstrated a typical Raman spectrum of single-layer graphene on the smooth Ti surface, with the G band at ~1580 cm^−1^ and a sharp and symmetric 2D band at ~2680 cm^−1^. And this is a typical spectrum for single-layer graphene distinguished from multilayer graphene. Therefore, we can conclude that single-layer graphene was successfully transferred to the Ti surface.

Meanwhile, the water contact angle and surface energy (Fig. 1D–F) demonstrated larger contact angle and lower surface energy of graphene-coated Ti samples compared with smooth Ti substrates, indicating that graphene-coated Ti was more hydrophobic than Ti substrate.

### Thermal treatment and adhesion strength of graphene sheets on Ti substrates

**Figure 2:**
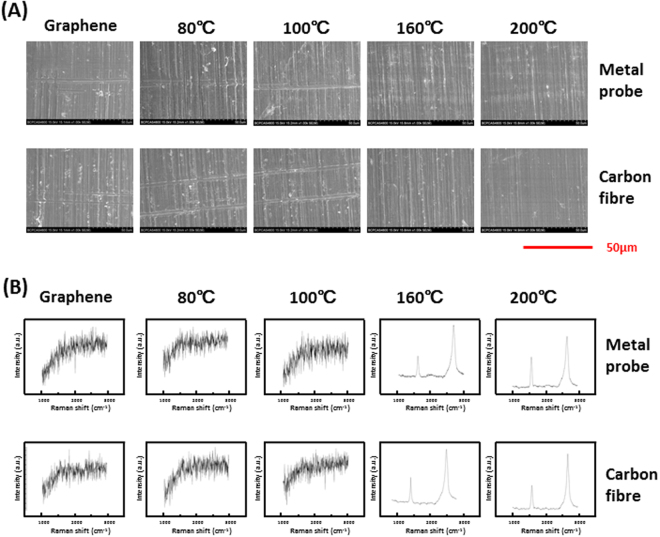
Dry heating treatment and tests of adhesion strength of graphene sheets on Ti substrates. (**A**) SEM images of scratched region of graphene and control surfaces after dry heating at different temperatures. (**B**) Raman spectra of scratched region of graphene and control surfaces after dry heating at different temperatures.

Dry heating treatment of 80, 100, 160, and 200 °C for 2 h was performed on graphene-coated Ti samples after the mediator-assisted transfer. Scratching tests with the tip of an ultrasonic scaler were performed to evaluate the adhesion of graphene on Ti substrates. Graphene sheets on Ti substrates after thermal treatment of 160 and 200 °C for 2 h remained intact after the scratching, while wrinkles or breakages were observed on graphene sheets after thermal treatment of 80 and 100 °C for 2 h (Fig. 2A).

Furthermore, we performed Raman spectroscopy of several points on each sample (with different temperatures of 80, 100, 160, and 200 °C for thermal treatment). By summarizing the results of these Raman spectroscopy examinations, we found that thermal treatment of 80, 100, 160, and 200 °C for 2 h did not influence the integrity of graphene sheets on Ti substrates, since the typical spectra of single-layer graphene could be observed on the area without scratches. On the other hand, the typical spectra of single-layer graphene still could be observed in the scratched areas of graphene coatings after thermal treatment of 160 °C and 200 °C (Fig. 2B), whereas graphene coatings were damaged in the scratched areas after thermal treatment of 80 °C and 100 °C (Fig. 2B). Therefore, thermal treatment of 160, and 200 °C for 2 h enhanced the adhesion between graphene and Ti substrate.

On the basis of these results, dry heating treatment at 160 °C for 2 h, a lower temperature than 200 °C, was identified as the optimal condition to achieve stronger adhesion between graphene and Ti substrates. Therefore, the following three groups were used in the subsequent experiments: titanium without graphene coating (No-Graphene); graphene-coated titanium (Graphene); graphene-coated titanium subjected to dry heating treatment at 160 °C for 2 h (Graphene-DH). As for surface characterization, there were no significant differences between the Graphene and Graphene-DH groups in roughness, water contact angle, and surface energy, indicating thermal treatment didn’t influence the physical characteristics of graphene (Fig. 1D–F).

### Adhesion and proliferation of hGFs, hASCs, and hBMMSCs

**Figure 3:**
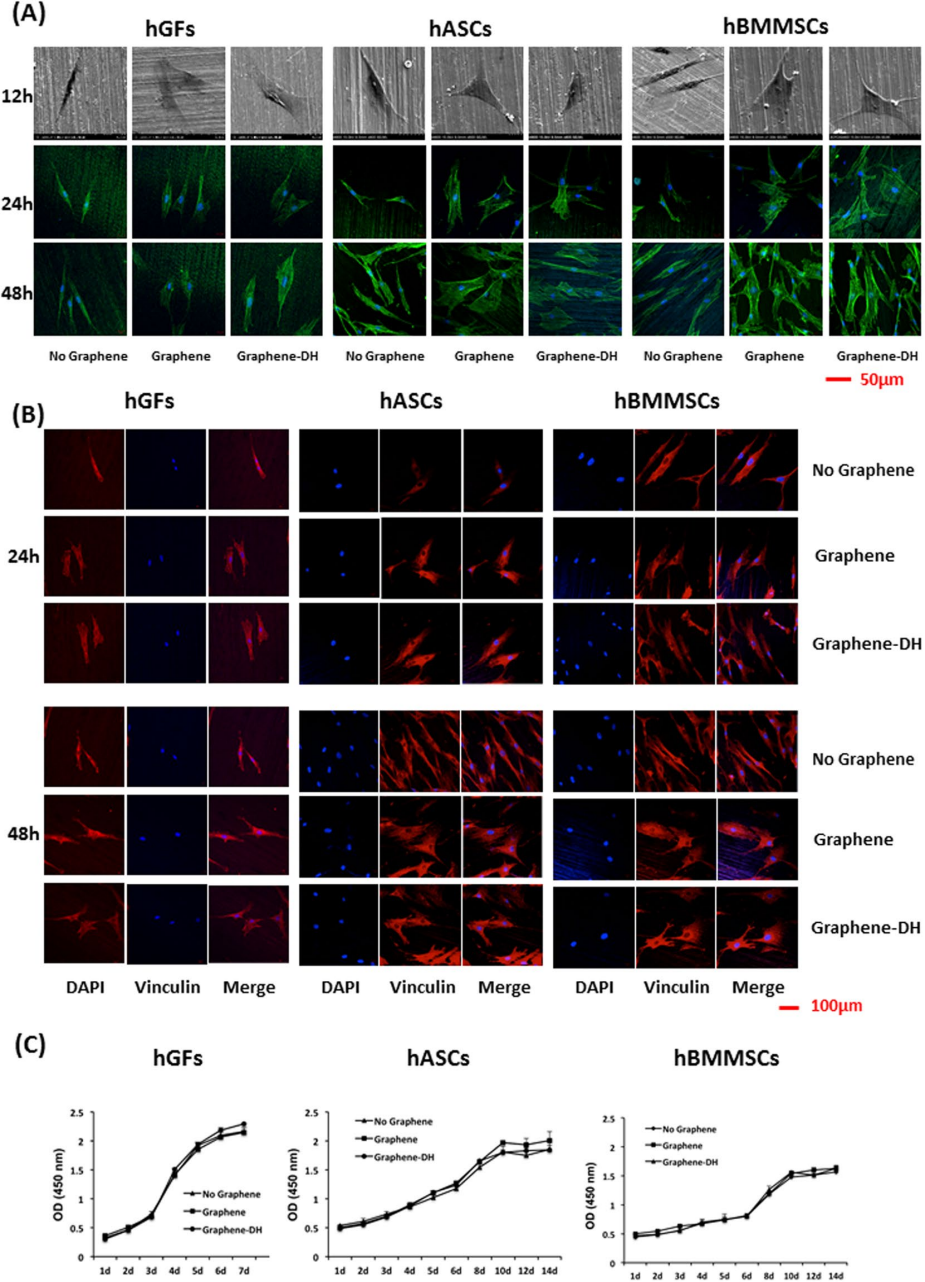
Adhesion and proliferation of hGFs, hASCs, and hBMMSCs on graphene. (**A**) SEM images and confocal micrographs of hGFs, hASCs, and hBMMSCs on graphene and control surfaces after 12, 24, and 48 h of culture. Phalloidin and nuclei are colored green and blue, respectively. (**B**) Immunofluorescent staining analysis of vinculin expression of hGFs, hASCs, and hBMMSCs on graphene and control surfaces after 24 and 48 h of culture. (**C**) CCK-8 assay of hGFs, hASCs, and hBMMSCs adhered on graphene and control surfaces.

SEM measurements and confocal microscopy images of FITC-phalloidin staining were used to characterize the morphology and fine structures of the adhered cells. After 12 h of culture, human gingival fibroblasts (hGFs), human adipose-derived stem cells (hASCs), and human bone marrow mesenchymal stem cells (hBMMSCs) on the Graphene and Graphene-DH samples presented obvious lamellipodia, while the pseudopodia of cells on the surfaces of uncoated Ti were relatively short and less evident (Fig. 3A). Meanwhile, more vinculin-positive tips of the pseudopodia were observed on Graphene and Graphene-DH groups than No-Graphene group (Fig. 3B).

The CCK-8 cell proliferation tests demonstrated similar logarithmic proliferation curves of hGFs, hASCs, and hBMMSCs on Graphene, Graphene-DH and No-Graphene surfaces (Fig. 3C).

### Osteogenic differentiation of hASCs and hBMMSCs

**Figure 4:**
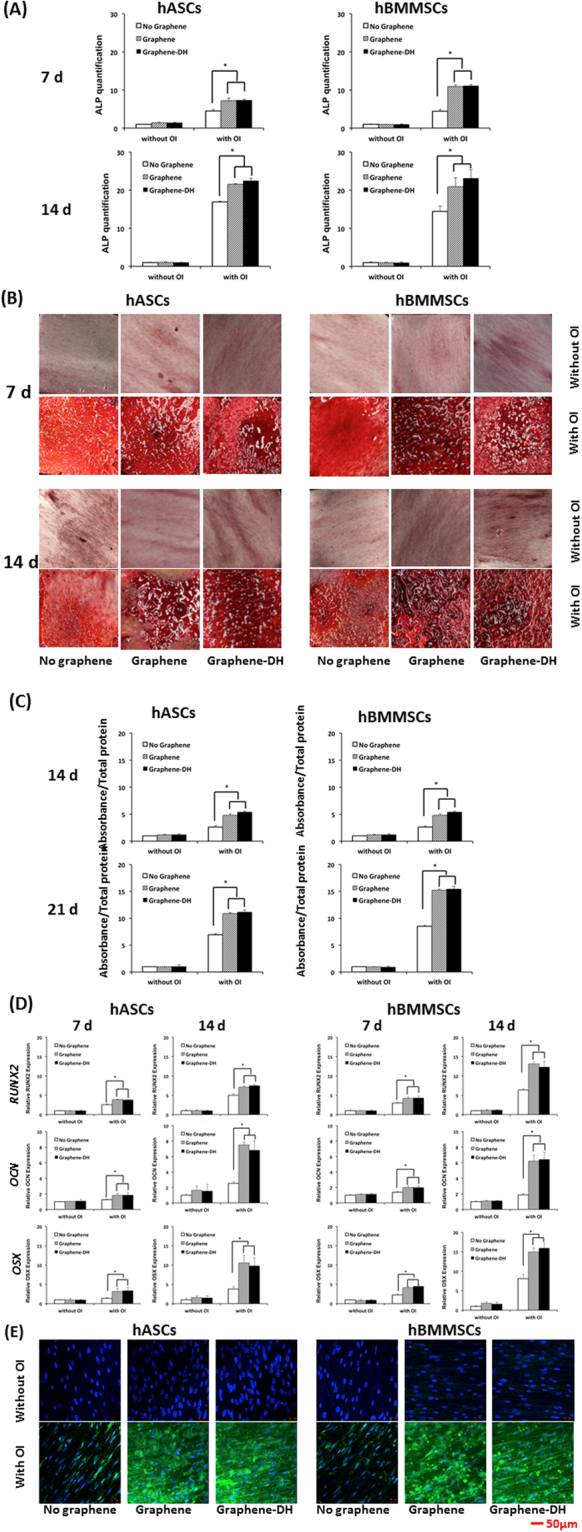
Osteogenic differentiation of hASCs and hBMMSCs on graphene *in vitro*. (**A**) ALP activity of hASCs and hBMMSCs cultured on graphene and control surfaces for 7 and 14 days. (**B**) Alizarin Red staining at 14 and 21 days. (**C**) Mineralization assay at 14 and 21 days. (**D**) Expression of osteogenic genes (*RUNX2*, *OCN*, and *OSX*) in hASCs and hBMMSCs cultured on graphene and control surfaces for 7 and 14 days. (**E**) Immunofluorescent staining analysis of OCN in hASCs and hBMMSCs cultured on graphene and control surfaces for 14 days. OCN and nuclei are colored green and blue, respectively. *P < 0.05.

Figure 4A showed that hASCs and hBMMSCs presented higher alkaline phosphatase (ALP) activity (*P* < 0.05) on the Graphene and Graphene-DH surfaces compared with the No-Graphene surfaces, whereas no significant differences could be observed between Graphene and Graphene-DH surfaces.

The alizarin red staining (AR-S) assays showed that, after 14 or 21 days of osteoinduction (OI), Graphene and Graphene-DH groups demonstrated stronger mineralization staining than No-Graphene for both hASCs and hBMMSCs, and there were no significant differences between Graphene and Graphene-DH groups (Fig. 4B). On the other hand, no obvious calcium nodule deposits were formed on any of the three groups without osteoinduction (Fig. 4B). Consistent with the AR-S staining, mineralization assays for both hASCs and hBMMSCs showed that specimens of the Graphene and Graphene-DH groups were more mineralized than those of the No-Graphene group (*P* < 0.05), and there were also no significant differences between Graphene and Graphene-DH groups (Fig. 4C).

After 7 and 14 days of osteoinduction, the expressions of osteogenesis-related genes, including *RUNX2*, osteocalcin (*OCN*), and Osterix (*OSX*), for both hASCs and hBMMSCs, were higher for Graphene and Graphene-DH groups than No-Graphene group (*P* < 0.05). Meanwhile, no significant differences were found between the Graphene and Graphene-DH groups. Moreover, there were no significant differences among the three groups when the cells were cultured without osteoinduction (Fig. 4D).

The expression of OCN protein was observed by immunofluorescence. Similar to the gene expression, after 14 days of OI, the Graphene and Graphene-DH groups presented more OCN-positive immunofluorescent staining than the No-Graphene group (Fig. 4E).

### Antibacterial activity *in vitro*

**Figure 5:**
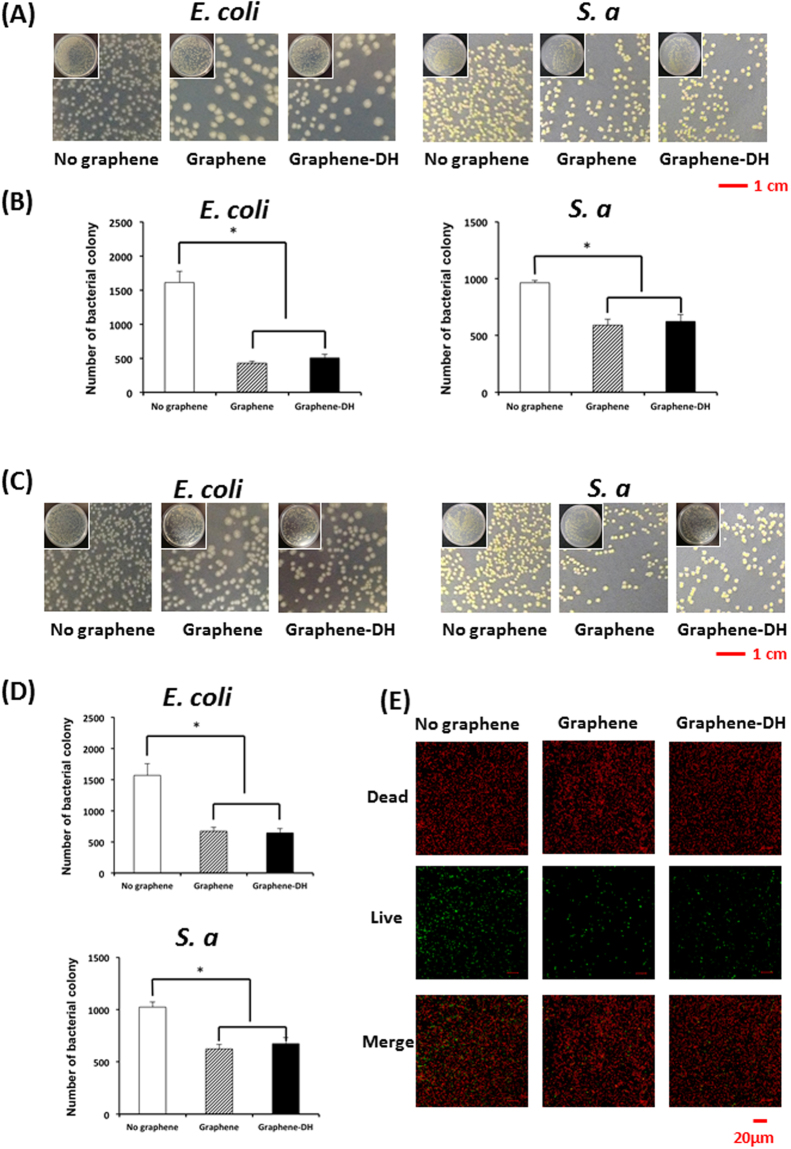
Antibacterial activity *in vitro*. (**A**) Photographs and (**B**) counting of bacterial colonies formed on graphene and control surfaces. (**C**)Photographs and (**D**) counting of bacterial colonies formed on graphene and control surfaces in repeated test after rinsing with PBS. (**E**) Fluorescence staining of viable bacteria colonization.

Bacterial counting was performed to investigate the response of Gram-negative *E. coli* and Gram-positive *S. aureus* bacteria to the three kinds of material surfaces examined in this study. Lower numbers of *E. coli* colonies were present on the Graphene and Graphene-DH surfaces compared with the No-Graphene group (*P* < 0.05), whereas no significant differences were observed between the Graphene and Graphene-DH samples (Fig. 5A,B). Lower numbers of bacterial colonies on the Graphene and Graphene-DH films were also observed after PBS rinsing and disinfection (Fig. 5C,D), indicating that the anti-bacterial ability of graphene can be maintained even after bacterial infection and disinfection by 75% alcohol. Similar results were observed for the *S. aureus* bacteria (Fig. 5A–D).

Fluorescence staining was used to visualize and to verify the anti-bacterial capability of the graphene films on viable bacteria colonization. After 24 hours of incubation, there were larger amounts of viable bacteria (green) on No-Graphene surfaces than on Graphene and Graphene-DH surfaces, whereas no significant differences were observed between Graphene and Graphene-DH samples (Fig. 5E). Meanwhile, there was no difference in the total amount of viable and dead bacteria among the three groups (Fig. 5E), indicating the antibacterial activity of graphene was achieved mainly by killing the attached bacteria, rather than preventing the attachment of the bacteria.

### Ectopic bone formation *in vivo*

**Figure 6:**
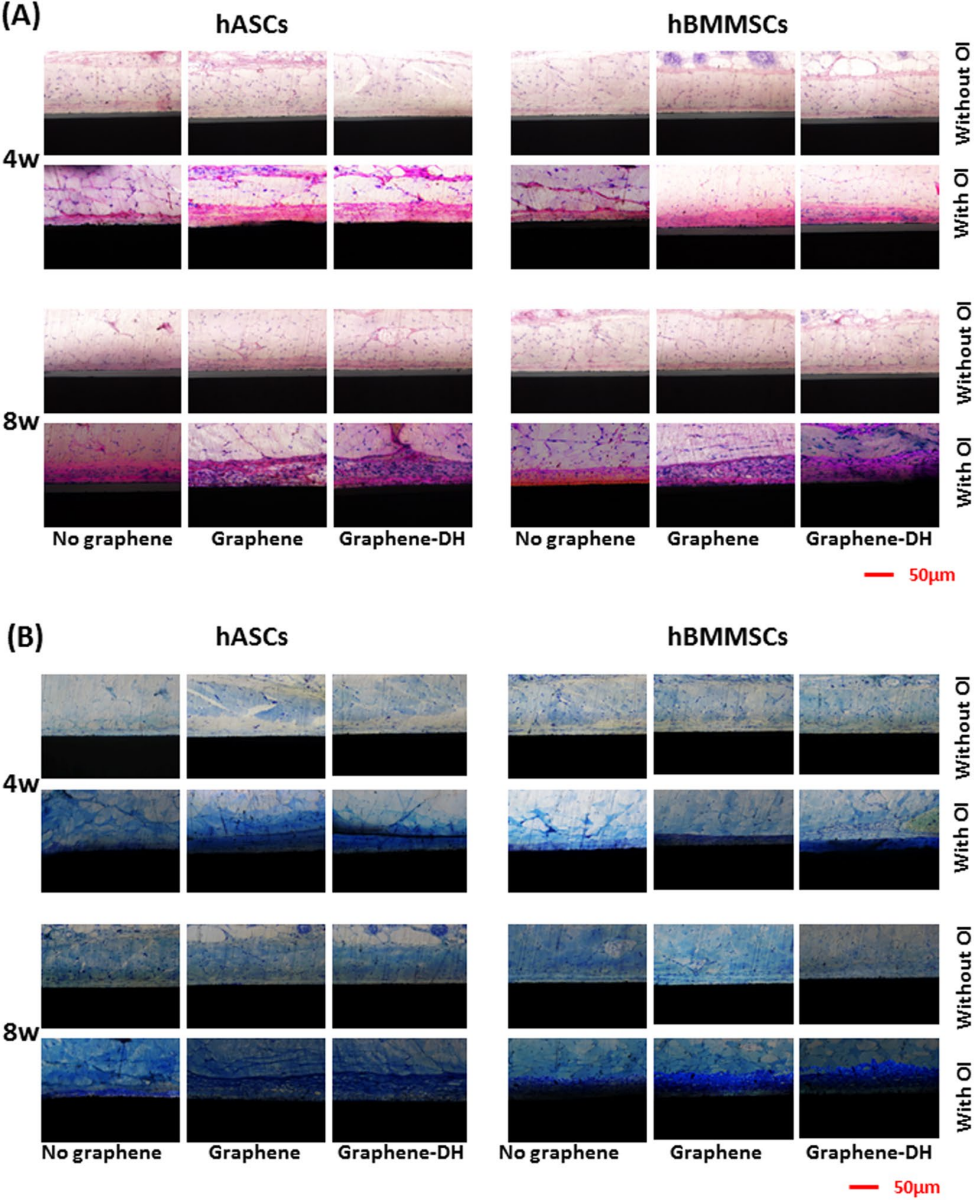
Ectopic bone formation *in vivo*. (**A**) HE and (**B**) toluidine blue staining on hard tissue slices four and eight weeks after implantation.

Four weeks after implantation, ectopic bone formation was evaluated by HE and toluidine blue staining. The bone matrix appeared as uniform acidophilic tissue after HE staining and had a dark blue appearance under toluidine blue staining. As shown in Fig. 6, the Graphene and Graphene-DH groups demonstrated increased bone matrix formation compared with the No-Graphene group. Although, the thickness of the new-born bone on the surfaces of Graphene and Graphene-DH groups was similar, the quality of the bone on Graphene-DH groups seemed better than Graphene groups with more acidophilic bone matrix after HE staining and more area of dark blue tissue after toluidine blue staining. However, no obvious bone matrix formation was detected in the three groups without osteoinduction.

Increased ectopic bone formation was observed for all groups at eight weeks after implantation, compared with that at four weeks, for both hASCs and hBMMSCs. The relative trends were the same as those observed after four weeks: the Graphene and Graphene-DH groups showed increased ectopic bone formation compared with No-Graphene (Fig. 6).

## Discussion

### Thermal treatment enhances the adhesion strength of graphene coatings on Ti substrates

Single-layer graphene can now be successfully produced in large scale by chemical vapor deposition on copper foil^3,31,32^. In this work, a mediator (PMMA)-assisted transfer technique was used to transfer the single-layer graphene on the surface of smooth titanium discs. Thermal treatment is the last step of the transferring process to remove PMMA by acetone steam bath for 30 min at 57 °C. In our previous research, we found that the adhesion strength between graphene and Ti was not strong enough for future clinical application. But interestingly, we found that prolonged time of thermal treatment improve the adhesion between graphene and Ti substrate. And this is where we got our enlightment to use thermal treatment to improve the adhesion strength between graphene and Ti.

The possible mechanism of enhanced adhesion strength between graphene and Ti after thermal treatment may result from further removal of residual PMMA and acetone, thus reducing the intervals between the graphene coatings and their underlying substrates. Uneven surfaces, such as wrinkles and ripples, are inevitable after transfer of single-layer graphene to other substrates. These areas are vulnerable due to higher density of intervals between the graphene coatings and the substrates, where microcracks will start and extend under the existence of external forces^34^.

As for the settings of temperatures for thermal treatment, we chose 80, 100, 160, and 200 °C, all higher than 56.53 °C (the boiling point of acetone), to remove the potential residual acetone. Meanwhile, the glass transition temperature of PMMA is around 150 °C. Therefore, we speculated that a thermal treatment with higher temperature than 150 °C might further remove the residual PMMA between graphene and titanium substrate, thus reducing the intervals between graphene coatings and Ti substrates. This is why 160 and 200 °C groups were chosen. By reducing residual acetone and PMMA by thermal treatment, a closer contact between graphene and the surface of titanium would be realized.

Scratch tests by a Florida Probe were used to reflect the adhesion strength between graphene sheets and the Ti substrates. With this special probe, the applied force could be kept constantly at 25 g, and therefore the comparability among the groups was assured. Ultrasonic scalers with carbon tips are widely used in periodontal maintenance therapy for dental implants. Therefore, we attached a carbon tip to the Florida Probe to scratch the surface of the materials, mimicking the clinical condition of periodontal probing and periodontal maintenance therapy of dental implants. After scratch tests, graphene coatings of Ti substrates after thermal treatment of 160 and 200 °C remained intact according to Raman spectra. 160 °C was identified as the optimal condition for thermal treatment to improve the adhesion strength of graphene coatings on titanium substrates because of the following reasons. Previous related studies showed that the thermal conductivity of graphene subjected to heat treatment at around 177 °C was similar to that measured around −3 °C^35^. Moreover, the thermal conductivity of graphene did not show significant changes between −173 and 127 °C^36^. Furthermore, Mounet *et al*. showed that the expansion coefficient of graphene remained substantially unchanged in the range of −23.15 to 127 °C^37^. The above studies demonstrated that the heat treatment of around 160 °C had no significant effects on the thermal conductivity and expansion coefficient of graphene. In this study, the AFM analysis of surface roughness and the water contact angle measurements demonstrated no differences between the Graphene and Graphene-DH samples. Therefore, thermal treatment at 160 °C is likely to exert no negative effect on the physical and chemical properties of graphene.

Meanwhile, dry heating treatment at 160 °C for 2 h is also the same condition as dry heating sterilization, a common and effective sterilization procedure applied in clinical practice^38^. Compared to autoclaving, dry heating sterilization reduces the rusting of metal medical materials^38^. Therefore, dry heating treatment at 160 °C for 2 h can not only enhance the adhesion strength of graphene on the Ti surface, but also combines the sterilization process, making it highly beneficial for clinical applications.

Most recently, a dry transfer technique based on a hot-pressing method was reported^39^, which may also be a potential transfer method for future clinical application of graphene-coatings on Ti substrates. However, the adhesion strength between graphene and Ti was not examined in the above study. Since the adhesion strength is a critical problem to be solved before the clinical application of graphene-coatings on Ti substrates, the examination of the adhesion strength is an indispensable index to evaluate new transfer methods. The scratch test, as proposed in this study, which used a Florida Probe to keep a constant force of 25 g, mimicking the clinical condition of periodontal probing and periodontal maintenance therapy of dental implants, is likely to be a practical method to evaluate the adhesion strength between graphene-coatings and their substrates.

### Effect of graphene-coated titanium on adhesion and proliferation of hGFs, hASCs, and hBMMSCs

The present results show that graphene promotes the adhesion of hASCs and hBMMSCs to the substrate. A stronger expression of vinculin was observed for the samples in the graphene-coated groups. Accordingly, the incorporation of graphene may provide a better substrate for the attachment of hASCs and hBMMSCs to titanium. In a previous report, Kim *et al*. investigated focal adhesion (FA) of hASCs on graphene-related materials and found higher numbers of FAs on graphene^40^. Furthermore, FAs were found to be more concentrated on the protruding ends of the cells in graphene films than in control groups^41^, in agreement with the present findings.

The oral environment around the neck of an implant is complex, and includes soft and bone tissue. Osseointegration determines the function and the survival life of the implants, and the good integrity of the implant neck and of the soft tissue around it is the key to a successful osseointegration. The loose contact between the titanium substrate and the surrounding soft tissue leads to microbial invasion, whose toxic products cause peri-implantitis. Therefore, adhesion and proliferation of hGFs were also examined in our study. Fortunately, we observed extended lamellipodia and higher numbers of vinculin positive tips of cell pseudopodia for hGFs on graphene-coated samples. These results suggest that the graphene coating on Ti substrates might improve the interaction between the material and the soft tissue around it.

Adhesion represents the crucial prerequisite for many cell functions, such as proliferation, synthesis of proteins, and formation of mineral deposits^42–44^. FAs are large protein complexes that regulate the connection between cells and extracellular matrix, and play a key role as mediators of cell adhesion; vinculin is a structural protein of FAs^45^. Several studies have suggested that the nanoscale structure of the substrate may regulate FAs^40^. We assumed that the nanoscale structure of graphene might promote the expression of FAs by regulating vinculin expression, thus influencing cell adhesion, and this may also play a role in other cell functions, including proliferation and osteogenic differentiation.

No significant differences in cell proliferation on the surfaces of the three types of investigated materials were observed, and it did not affect the growth of hGFs, hASCs, and hBMMSCs. This was consistent with the results of researches on single-layer graphene^8,46^, and at the same time graphene did not show positive effects on cell proliferation reported in other studies with multilayered graphene and graphene oxide^41,47,48^. This suggests that the introduction of graphene did not affect the physiological cell growth conditions. In addition, the treatment at 160 °C for 2 h did not alter the effect of graphene on cell adhesion and proliferation.

### Antibacterial effect of graphene-coated titanium

Since the discovery of the antibacterial effects of a graphene oxide suspension in 2010, a large number of studies have shown that graphene oxide nanosheets and its derivatives have marked antibacterial properties^49–51^. Some of them reported that graphene oxide almost completely suppressed the growth of bacteria, leading to a viability loss up to 80–90%^49^. However, only few studies examined the antibacterial activity of single-layer graphene. In 2004, Li *et al*. reported the antibacterial properties against *E. coli* and *S. aureus* of single-layer graphene on different substrates including Cu, Ge, and SiO_2_, and found that graphene on Cu had the best antibacterial ability among these substrates, followed by graphene on Ge and graphene on SiO_2_^16^. However, Dellieu *et al*. proposed a different result^52^. The divergence was mainly resulted from lack of exact viability loss rate of the bacteria. In this study, we tested the antibacterial activity of graphene on a titanium substrate, and demonstrated its *in vitro* activity against *E. coli* and *S. aureus* bacteria. Meanwhile, fluorescence staining demonstrated significantly lower amounts of viable bacteria on the Graphene and Graphene-DH surfaces, indicating that the anti-bacterial activity of graphene was achieved mainly by killing the attached bacteria, rather than preventing the attachment of bacteria. Moreover, thermal treatment of 160 °C for 2 h did not affect the antibacterial activity of graphene on the titanium discs.

Huang *et al*. proposed that the antibacterial activity of graphene is due to the mechanical destruction of the cell membrane caused by the sharp edges of the graphene structure^12^. Subsequent studies suggested that oxidative stress mediated by superoxide ions might play a role in the antibacterial effects of graphene^15,16,53^. Recently, Tu *et al*. reported the destructive extraction of phospholipids from the molecular cell membrane upon interaction with graphene validated by the TEM images, which could be one of the mechanisms behind its antibacterial effects^54^. This strong attraction between graphene and membrane lipids is largely derived from graphene’s unique two-dimensional structure with all sp^2^ carbons, which facilitates exceptionally strong dispersion interactions between graphene and lipid molecules.

Interestingly, the destructive effect of graphene on the membrane of bacterial cells is cell-specific: for instance, graphene has little effects on mammalian cells. This may be because the abundant glycoproteins on the surface of the mammalian cell membrane block out the surface of graphene and protect the phospholipids from adsorption. Basing on the information our tests for antibacterial activity of graphene and recent related studies, knowledge on the exact mechanism is still limited. Since bacterial species involved in the studies are finite, whether graphene has long-term and broad-spectrum antibacterial effects still needs to be further confirmed. Moreover, whether the interaction between single-layer graphene and bacteria is similar to that of graphene oxide or other graphene-related material need to be further explored.

### Enhanced osteogenic differentiation of MSCs by graphene-coated Ti

In this study, we demonstrated that graphene-coated titanium can promote the osteogenic differentiation of hASCs and hBMMSCs *in vitro* and *in vivo*, and showed that a heat treatment does not influence these favorable effects. In this research, we found that gene expressions of osteogenic-related genes, such as *RUNX2 OSX* and *OCN*, and the protein expression of OCN, were upregulated by graphene under the existence of OM, but the osteogenic differentiation of mesenchymal stem cells on graphene without OM was not significantly promoted compared with Ti substrate. However, some existing studies^46,55,56^ demonstrated the so-called “spontaneous osteogenic differentiation”, referring to the phenomenon that graphene promotes osteogenic differentiation without the existence of osteoinducing factors. These different results were likely to be caused by difference between the substrate under graphene. The substrates used these researches were glass or Si. But we used Ti as substrate in this research. As we know, Ti is the most widely used material is bone implant due to its good biocompatibility and advantages in osteointegration. Therefore, graphene didn’t accelerate the osteogenic differentiation so much compared with Ti surface without the existence of osteoinducing factors. However, under the existence of osteoinducing factors, graphene demonstrated a synergystic effect with OM as we discovered in this article. This phenomenon may result from preconcentration of osteogenic inducers, such as dexamethasone and β-glycerolphosphate, due to graphene’s strong π–π stacking, hydrogen bonding, and electrostatic interactions with proteins.

Previously, a number of studies have demonstrated that single-layer graphene can improve the osteogenic differentiation of mesenchymal stem cells (MSCs) and osteoblasts^46^. Meanwhile, multilayer graphene^57^, graphene oxide^10,40,58^, and graphene-related composites^59^ can improve the osteogenic differentiation of mesenchymal stem cells (MSCs) and osteoblasts^60,61^. Here, we showed that graphene maintained its positive effects on *in vitro* and *in vivo* osteogenic differentiation of hASCs and hBMMSCs after being transferred to titanium and heated at 160 °C for 2 h. Meanwhile, as for *in vivo* study, although the thickness of the new-born bone on the surfaces of Graphene and Graphene-DH groups seemed similar, the quality of the bone on Graphene-DH groups was better than Graphene groups with more acidophilic bone matrix after HE staining and more area of dark blue tissue after toluidine blue staining. This may result from better adhesion strength between graphene-coatings and Ti substrates, which helped to maintain the integrity of graphene-coatings even under sustained mechanical interferences from *in vivo* environment. Clearly, this conclusion is limited to the animal models employed in our study, and graphene-coated titanium bone implants and *in situ* osteointegration^62^ will be a practical *in vivo* model to illustrate the future application of graphene-coated Ti implants. Basing on the methods to enhance and evaluate the adhesion strength of graphene-coatings on Ti as proposed in this study, we will uncover the exact effect of bone integration of graphene-coated titanium bone implants *in vivo* by using the *in situ* osteointegration model in our future experiments.

The mechanism leading to the favorable effect of graphene on osteogenic differentiation has been previously explored from several angles, including morphological^10,41,46^, mechanical^48^, biochemical, and molecular aspects^10,60^. We previously reported the effect of graphene on cell behavior from the viewpoint of epigenetic regulation, and revealed that graphene promotes osteogenic differentiation of hMSCs by upregulating the methylation level of H3K4 at the promoter regions of osteogenesis-associated genes by inhibiting the RBP2 expression^13^. Thus, multiple factors may play a role in the graphene-cell interactions, and its internal mechanisms remain to be explored.

In this study, we managed to modify titanium by using the anti-bacterial and osteoinductive effects of single-layer graphene sheet. Despite the satisfactory results obtained a number of issues still need to be solved before its application. First, the stability of the graphene-titanium association during clinical operation remains to be tested. Second, the exact mechanism of graphene-cell and graphene-tissue interactions is still unclear. Finally, further studies are needed to determine the efficiency and range of the graphene antibacterial activity in the complex oral environment.

## Conclusions

Thermal treatment at 160 °C for 2 h could enhance the adhesion strength between graphene coatings and titanium substrates. Graphene coatings enhanced the adhesion of hGFs, hASCs, and hBMMSCs, and promoted the *in vitro* and *in vivo* osteogenic differentiation of hASCs and hBMMSCs. In addition, graphene coatings enjoy superior antibacterial effects. Thermal treatment will not influence the favorable effects of graphene on cell adhesion, osteogenic differentiation and antibacterial ability. Therefore, we managed to incorporate both the antibacterial and osteoinductive effects of single-layer graphene sheets on titanium substrates, and facilitate the future clinical application by enhancing their adhesion strength by thermal treatment.

## Methods

### Ethics statement

Our research was approved by the Ethics Committee of the Peking University Health Science Center, Beijing, China (PKUSSIRB-2013023). All *in vivo* experiments complied with the ARRIVE guidelines and were carried out in accordance with the U.K. Animals (Scientific Procedures) Act, 1986 and associated guidelines, EU Directive 2010/63/EU for animal experiments.

### Preparation of smooth and graphene sheet-coated Ti discs

Single-layer graphene grown on copper foil substrate by CVD (purchased from American Chemical Society, ACS) and smooth Ti discs (99.6% purity, Leiden, Beijing, China) were cut into appropriate sizes. Ti discs were polished with silicon carbide sandpaper of No. 240, 360, 400, 600, 800, 1000 and 2000 grits in series, and then washed with acetone, absolute alcohol and deionized water (dH_2_O) (Milli-Q Ultra-Pure, Millipore, Billerica, MA, USA) in an ultrasonic cleaner, respectively, for 15 min. Subsequently, the specimens were dried at room temperature for 1 h. For the transfer process, a thin film of PMMA (950 K grade, 2 wt% in chlorobenzene) was spin-coated on graphene by a Laurell^®^ WS-400BZ-6NPP/LITE spin coater. After treatment at 180 °C for 4 min, the PMMA-coated graphene was transferred to a FeCl_3_ solution (0.05 g/mL in water) to remove the copper foil substrate, and rinsed with distilled water twice to remove the residues. A smooth Ti disc was then placed in distilled water underneath the film and was picked up from the water with the coating of the film. Finally, the thin PMMA film on the top of graphene was removed by immersion in an acetone steam bath for 30 min at 57 °C. Atomic force microscopy (AFM) measurements and Raman spectra with an excitation laser source of 532 nm were used to confirm the presence of single-layer graphene on the Ti discs. Water contact angles, measured by a SL200 system (Kino Industry, New York, USA), were used to examine the surface characteristics of the Ti discs coated with graphene. Before using them for the *in vitro* and *in vivo* experiments, the graphene-coated Ti discs were disinfected by soaking in 75% alcohol for 30 min.

### Thermal treatment and analysis of adhesion strength

After the transfer process, the graphene-coated Ti discs were treated at 80, 100, 160, and 200 °C in a vacuum drying oven for 2 h. The Florida Probe (Florida Probe Corporation, FL, USA) was used to inspect the adhesion of graphene on Ti substrate. With this special probe, the applied force could be kept constant at 25 g. We attached a carbon tip (Premier Dental Products, Ontario, Canada) to the Florida Probe to scratch the surfaces of graphene-coated Ti, mimicking the clinical condition of periodontal probing and periodontal maintenance therapy of dental implants. Both scratched and unscratched areas were analyzed by field-emission scanning electron microscopy (FESEM, Hitachi S4800, Japan) and Raman spectroscopy to evaluate the integrity of graphene coatings.

### Culture and osteogenic induction of hASCs, hBMMSCs, and hGFs

hASCs and hBMMSCs were purchased from ScienCell Research Laboratories (San Diego, CA, USA), whereas hGFs were obtained from the attached gingiva of human premolars. The cells were cultured in low-glucose Dulbecco’s modified Eagle medium (DMEM) supplemented with 10% fetal bovine serum (FBS), 100 U/mL penicillin, and 100 mg/mL streptomycin for proliferation. DMEM, FBS, 100 U/mL penicillin, and 100 mg/mL streptomycin were purchased form Gibco (Grand Island, NY, USA). For the osteogenic differentiation, 10 nM dexamethasone, 10 mM *β*-glycerophosphate, and 50 μg/mL l-ascorbic acid were added to the medium. Cells were cultured in a controlled environment at 37 °C in an incubator (95% air, 5% CO_2_, 100% relative humidity). All subsequent *in vitro* and *in vivo* experiments were performed using cells at the third and fourth passage. Moreover, all experiments were carried out in triplicate with cells extracted from three different patients.

### Adhesion and proliferation assays

The hASCs, hBMMSCs, and hGFs cells were seeded on titanium substrate (No-Graphene), titanium coated with graphene (Graphene), and titanium coated with graphene after dry heating treatment (Graphene-DH), at 10,000 cells/well in a 24-well plate, in a normal stem cell medium. After 12 h of incubation, the cells were washed three times with phosphate buffered saline (PBS) and fixed in cacodylate-buffered 4% glutaraldehyde at of 4 °C for 12 h. Then, the samples were dehydrated under a series of ethanol solution and dried in a professional dryer (Micro Modulyo 230, Thermo Scientific, Waltham, MA, USA). After coating with gold, the specimens were observed by SEM (Hitachi S4800, Japan). After 24 and 48 h of incubation, the cells were washed three times with PBS and fixed in 4% paraformaldehyde for 20 min. Then, after postfixing in 0.1% Triton X-100 for 5 min at room temperature, the cells were incubated in fluorescein isothiocyanate (FITC)-labeled phalloidin for 25 min to stain the cytoskeleton and in 6-diamidino-2-phenylindole (DAPI) solution for 10 min to stain the nucleus. The stained cells were observed by a Confocal Zeiss Axiovert 650 microscope (Carl Zeiss Microimaging, LLC, Thornwood, NY, USA), using excitation laser wavelengths of 488 and 405 nm.

CCK-8 tests were carried out to monitor the proliferation of hASCs, hBMMSCs, and hGFs. Each day during two weeks of incubation, cells in each group were incubated with the counting reagent for 3 h, according to the manufacturer’s instructions. The relative cell number was determined by measuring the light absorbance (optical density, OD) at 450 nm of the formazan dye product in the cultures^63^.

### Alkaline phosphatase (ALP) activity of hASCs and hBMMSCs on graphene

To test the ALP activity, hASCs and hBMMSCs were seeded on different surfaces at 10,000 cells/well in 24-well plates. The ALP activity of each group was determined after osteoinduction (OI) for 7 and 14 days. The ALP levels were normalized to the total protein content, as previously described^64^.

### Alizarin red S (AR-S) staining and mineralization assays

hASCs and hBMMSCs were seeded onto different surfaces under the same conditions described above (10,000 cells/well, 24-well plate). After 14 and 21 days of osteoinduction, cells on different surfaces were stained with AR-S to monitor the mineralization. The specimens were rinsed three times with PBS and fixed in ice-cold 70% ethanol for 30 min at room temperature, then stained with 0.5% AR-S solution for 1 h to stain the calcium deposits. Finally, the samples were rinsed three times with distilled water.

To quantify matrix mineralization, the AR-S-stained samples were incubated in 100 mM cetylpyridinium chloride for 1 h to solubilize and release calcium-bound AR-S into the solution. The absorbance of the released AR-S was measured at 562 nm. The final calcium levels in each group were normalized to the total protein concentrations obtained from duplicate plates.

### RNA extraction, reverse transcription, and quantitative real-time polymerase chain reaction (PCR) analysis

hASCs and hBMMSCs were seeded on different surfaces as described above, in 6-well plates. After 7 and 14 days of osteoinduction, the Trizol reagent (Invitrogen, Carlsbad, CA, USA) was used to isolate the total cellular RNAs of each group. After synthesizing the first strand cDNA using the reverse transcription system (Roche, Basel, Switzerland), quantification of all gene transcripts was performed by real-time polymerase chain reaction (qPCR) using a Power SYBR Green PCR Master Mix and an ABI PRISM 7500 sequence detection system (Applied Biosystems, Foster City, CA, USA). The *β*-actin expression was used as the internal control. The primer sequences were shown in Table 1. The cycle threshold values (Ct values) were used to calculate the fold differences among the samples, using the ∆∆Ct method^65,66^.

**Table 1:** Primers for realtime PCR.

Gene	Forward primers	Reverse primers
*RUNX2*	ATGGGATGGGTGTCTCCACA	CCACGAAGGGGAACTTGTC
*OSX*	CCTCCTCAGCTCACCTTCTC	GTTGGGAGCCCAAATAGAAA
*OCN*	CACTCCTCGCCCTATTGGC	CCCTCCTGCTTGGACACAAAG
β-actin	CATGTACGTTGCTATCCAGGC	CTCCTTAATGTCACGCACGAT

### Immunofluorescence tests

The specimens were rinsed three times in PBS for 5 min and fixed in 4% paraformaldehyde for 20 minutes at room temperature. The samples were washed another three times in PBS and were incubated with specific primary antibodies at 4 °C for 12 h. Anti-vinculin (1:200) and anti-osteocalcin (1:500) primary antibodies (Santa Cruz, Dallas, TX, USA) were used to monitor the expression of vinculin and osteocalcin, respectively. The specimens were rinsed another three times in PBS and incubated in 1:500 anti-rabbit or anti-mouse secondary antibodies (4412S, 4528S, Cell Signaling Technology, Beverly, MA, USA) for 1 h at room temperature. Afterward, the specimens were stained in DAPI solution for 10 min at 37 °C before being visualized with a Confocal Zeiss Axiovert 650 microscope (Carl Zeiss Microimaging, Oberkochen, Germany), using laser wavelengths of 405, 488, and 543 nm.

### Evaluation of antibacterial activity

The antibacterial activity of the samples was assessed using Gram-negative *E. coli* and Gram-positive *S. aureus*. Following Li *et al*.’s approach^14^, a 10^4^ CFU/mL bacteria solution was introduced to the samples at a density of 80 μL/cm^2^. After inoculation at 37 °C for 24 h, the dissociated bacteria solution was collected and inoculated into a standard agar culture medium. After incubation at 37 °C for 24 h, the culture plates were photographed and the colony forming units were counted. The samples (Graphene, Graphene-DH, and No-Graphene) inoculated with bacteria were then rinsed three times with PBS, and disinfected in 75% alcohol for 30 min. Later, a same density of 10^4^ CFU/mL bacteria solution was introduced to the samples at a density of 80 μL/cm^2^ as described above, to examine whether the antibacterial activity of graphene-coated Ti could still exist after bacterial inoculation and disinfection.

The antibacterial activity of the graphene samples was evaluated by using Gram-negative *E. coli*. In order to visualize the viability of bacteria on the samples, a LIVE/DEADH BacLightTM Bacterial Viability Kit (L13152, Molecular Probes) was used, which contained the green fluorescent DNA-binding stain, Syto 9 and the red fluorescent DNA-binding stain, propidium iodide (PI), SYTO 9 permeated both intact and damaged membranes of the cells, binding to nucleic acids and fluorescing green when excited by a 485 nm wavelength laser, but PI entered only cells with significant membrane damage, which are considered to be non-viable^67^. The staining procedure was carried out according to the manufacturer’s protocol, bacteria at a concentration of 10^7^ CFU/mL were inoculated on the samples. After incubation at 37 °C for 24 h^16^, the culture medium was removed and the samples were rinsed with deionized water, then the staining solution was added. After being incubated in the dark at room temperature for 15 min, the samples were observed by Confocal laser scanning microscopy.

### Ectopic bone formation *in vivo*

To determine the effect of graphene on bone formation *in vivo*, hASCs and hBMMSCs were cultured on different specimens and then implanted into the dorsal subcutaneous area of eight week-old male BALB/c nude mice, according to the procedure described previously^44^. For each samples (No graphene, Graphene, and Graphene-DH), the following test groups were examined: discs incubated with hASCs in proliferation medium (hASCs without OI), discs incubated with hASCs in osteoinducing medium (hASCs with OI), discs incubated with hBMMSCs in proliferation medium (hBMMSCs without OI), discs incubated with hBMMSCs in osteoinducing medium (hBMMSCs with OI). After four and eight weeks of normal diet, the implants of each group were harvested together with the surrounding tissues. Following fixation with formalin and resin infiltration, the implants were stained with hematoxylin/eosin (HE) and toluidine blue. Bone formation was then observed with a light microscope.

### Statistical analysis

All results are presented as mean ± standard deviation; the data were analyzed using the SPSS 19.0 software (SPSS Inc., Chicago, IL, USA) by one-way ANOVA followed by a Tukey’s *post hoc* test. For all tests, *P*-values less than 0.05 were considered indicative of statistically significant differences.
